# Comparison of the Effects of Sevoflurane and Desflurane on Endothelial Glycocalyx in Patients Undergoing Laparoscopic Hysterectomy: A Randomized, Double-Blind Trial

**DOI:** 10.4274/TJAR.2024.231323

**Published:** 2024-05-03

**Authors:** Kemal Tolga Saraçoğlu, Tahsin Şimşek, Hande Gürbüz, Fatih Doğu Geyik, Ebru Kale, Kürşad Nuri Baydili, Raghad Giuma M. Kordi, Ahmet Kale, Ayten Saraçoğlu

**Affiliations:** 1 Qatar University, Hazm Mebaireek General Hospital HMC, Department of Anaesthesiology, Intensive Care Unit and Perioperative Medicine, Doha, Qatar; 2 University of Health Sciences Turkey, Kartal Dr. Lütfi Kırdar Kartal City Hospital, Clinic of Anaesthesiology and Reanimation, İstanbul, Turkey; 3 Bursa School of Medicine, Bursa City Hospital, Clinic of Anaesthesiology and Reanimation, Bursa, Turkey; 4 University of Health Sciences Turkey, Hamidiye Faculty of Medicine, Department of Biochemistry, İstanbul, Turkey; 5 University of Health Sciences, Management and Organization Program, İstanbul, Turkey; 6 Marmara University Faculty of Medicine, Department of Anaesthesiology and Reanimation, İstanbul, Turkey; 7 University of Health Sciences Turkey, Kartal Dr. Lütfi Kırdar Kartal City Hospital, Clinic of Obstetrics and Gynecology, İstanbul, Turkey; 8 Qatar University, Aisha Bint Hamad Al Attiyah Hospital HMC, Department of Anaesthesiology, Intensive Care Unit and Perioperative Medicine, Doha, Qatar

**Keywords:** Desflurane, endothelium, glycocalyx, heparan sulfate proteoglycans, sevoflurane, syndecans

## Abstract

**Objective:**

Various enzymes, reactive oxygen species, inflammatory conditions, and major surgeries cause endothelial glycocalyx breakdown. Inhalation of anaesthetic agents may have protective effects on the endothelium. This study compared syndecan-1 and heparan sulfate levels to evaluate the effects of sevoflurane and desflurane on the endothelial glycocalyx.

**Methods:**

This prospective randomized, double-blind study included 46 patients undergoing laparoscopic hysterectomy. The participants were allocated into sevoflurane and desflurane groups. Subsequently, blood samples were drawn at three time points: before anaesthesia induction for a baseline value (T0), after pneumoperitoneum (T1), and after extubation (T2). Heparan sulfate and syndecan-1 levels were measured.

**Results:**

There was no statistical difference between the sevoflurane and desflurane groups in terms of heparan sulfate and syndecan-1 levels at any time point. A significant difference was found only in the desflurane group in the intragroup comparisons of the measurements of heparan sulfate levels (χ^2^=29.826, *P* < 0.001). Matched pairs of the time points in the desflurane group showed that *P*=0.036 (Z=-2.099) for T1-T0, *P* < 0.001 (Z=-3.924) for T2-T0, and *P* < 0.001 (Z=-4.197) for T2-T1. The change in percentage between T2 and T1 of heparan sulfate in the desflurane group was found to be statistically significant (*P*=0.034).

**Conclusion:**

The damage caused by surgical stress on the endothelial glycocalyx can be reduced by both desflurane and sevoflurane. The protective effect of desflurane is more prominent than that of sevoflurane.

**Main Points**

• The endothelial glycocalyx is a crucial element of endothelial function.

• Disrupting endothelial glycocalyx integrity results in interstitial edema and coagulation disorders.

• Inhalation agents prevent endothelial glycocalyx degradation compared with intravenous anaesthetics.

• The damage caused by surgical stress on the endothelial glycocalyx can be reduced by both desflurane and sevoflurane.

• The protective effect of desflurane is more prominent than that of sevoflurane.

## Introduction

The glycocalyx is a dynamic and complex biochemical structure that consists of glycosaminoglycans (hyaluronic acid, heparan sulfate, chondroitin sulfate), glycoproteins (syndecan), and plasma proteins (albumin, antithrombin-III).^1^ The endothelial glycocalyx forms a thick and physiologically active layer on the vessel surface that regulates oncotic pressure and prevents leukocyte and platelet adhesion to the endothelium.^2,3^ Glycocalyx damage causes increased fluid permeability, resulting in interstitial edema and increased leukocyte and platelet adhesion, resulting in coagulation disorders.^4,5^ Various enzymes, reactive oxygen species, inflammatory conditions, severe multiple traumas, and major surgical procedures lead to alterations in the endothelial glycocalyx.^6^

Compared with laparotomic surgery, laparoscopic surgery causes less surgical trauma, stress, and inflammatory response.^7^ However, abdominal distension during pneumoperitoneum, which is necessary to create sufficient vision and working space in laparoscopic surgery, may also decrease splanchnic blood flow and organ ischemia. Moreover, deflation of the pneumoperitoneum may cause ischemia-reperfusion injury and oxidative stress.^8,9^ These conditions, which are highly associated with endothelial glycocalyx degradation, can cause adverse outcomes.^6,9^

Hydrocortisone and anti-TNF-α agents help reduce surgical stress-induced mediator release and inflammation.^10^ In addition, although inhalational and intravenous anaesthetic agents are thought to have protective effects against endothelial glycocalyx damage, previous studies have produced conflicting results. For instance, sevoflurane is effective in preventing endothelial glycocalyx degradation compared with controls.^11,12^ However, neither sevoflurane nor desflurane was found to be superior to propofol in protecting the endothelial glycocalyx.^13,14,15^ In contrast, another study suggested that sevoflurane resulted in lower syndecan-1 and heparan sulfate levels than propofol.^16^

Based on the existing literature, no studies have compared the effects of sevoflurane and desflurane on the endothelial glycocalyx. With the hypothesis that the effects of sevoflurane and desflurane on endothelial glycocalyx may vary depending on the differences in their chemical structures, we aimed to compare the serum syndecan-1 and heparan sulfate levels of patients undergoing laparoscopic hysterectomy under sevoflurane- or desflurane-based anaesthesia.

## Methods

### Ethics

Ethical approval of the study protocol was provided by the University of Health Sciences Turkey, Kartal Dr. Lütfi Kırdar Kartal City Hospital, Clinical Research Ethics Committee with protocol #514/192/32 and dated December 30, 2020. The trial was registered on www.clinicaltrials.gov with the reference NCT05068336. Written informed consent for participation was obtained from all patients before the trial. The study was conducted in accordance with the Declaration of Helsinki and reported in adherence to the CONSORT guidelines for randomized trials.

### Study Design

This prospective, randomized, double-blind study was conducted at a university hospital between August 15, 2021, and November 5, 2021. The primary outcome was to compare serum syndecan-1 and heparan sulfate levels with sevoflurane- and desflurane-based anaesthesia. The secondary outcome was to explore the variation in syndecan-1 and heparan sulfate levels.

### Participants

Patients scheduled for elective laparoscopic hysterectomy who were 18-65 years old with American Society of Anesthesiologists I and II were asked to participate in the trial. The predetermined exclusion criteria were refusal to participate in the study, risk of malignant hyperthermia, difficult airway, need for blood product transfusion, emergency surgery, and conversion from laparoscopy to laparotomy for any reason.

### Anaesthesia Protocol

All participants received a standard anaesthesia regimen. Standard monitoring techniques, including electrocardiogram, pulse oximetry, end-tidal carbon dioxide, and noninvasive blood pressure, were applied to the patients in the operating room. A bispectral index monitor was not used. Body temperature was measured using an esophageal probe, and all patients were heated intraoperatively with a warming blanket. No premedication was administered. After establishing intravenous access, standard anaesthesia induction was performed with 1-1.5 mg kg^–1^ propofol, 1-2 µg kg^–1^ fentanyl, and 0.6 mg kg^–1^ rocuronium (lidocaine was not used). After endotracheal intubation, general anaesthesia was maintained with the inhalational anaesthetics sevoflurane or desflurane (according to the allocation) in 2 L min of 50-50% oxygen-air mixture. For the maintenance of anaesthesia, sevoflurane at a 2-2.5% concentration or desflurane at a 6-8% concentration was used to maintain a minimum alveolar concentration of 1. The patients were ventilated in the pressure-controlled, volume-guaranteed mode, with the tidal volume set at 8 mL kg^-1^ of ideal body weight.

All patients received a standard fluid treatment protocol. Crystalloids (ringer lactate) were infused at a rate of 1-1.5 mL kg^-1^ h^-1^ during surgery. No colloids were used. Tramadol (100 mg) and paracetamol (1 g) were administered for postoperative analgesia to all patients 20 min at the end of the surgery. At the end of the surgery, the neuromuscular blocker was antagonized with the administration of 0.015 mg kg^-1^ atropine and 0.04 mg kg^-1^ neostigmine. After extubation, the patients were transferred to the post-anaesthesia care unit following adequate muscle strength and spontaneous ventilation.

### Collection of Blood Samples

Venous blood samples to analyze syndecan-1 and heparan sulfate levels were drawn at three time points: intravenous access placement in the operating room, before anaesthesia induction for a baseline value (T_0_), 5 min after gas insufflation for pneumoperitoneum but before the initiation of surgery (T_1_), and 5 min after extubation of the endotracheal tube at the end of surgery (T_2_). Blood samples were placed in a 5 mL vacuum tube. The samples were immediately centrifuged at a rate of 1500 × g for 10 min at 4 °C to separate the serum, and the supernatant was stored at 80 °C for further analysis of syndecan-1 and heparan sulfate.

### Biochemical Analysis

Studies for the analysis of heparan sulfate and syndecan-1 parameters as indicators of endothelial glycocalyx injury were performed at the institutional medical biochemistry laboratory. The serum concentrations of these indicators were measured with the enzyme-linked immunosorbent assay (ELISA) technique using commercial ELISA kits (BT-Lab, Shanghai Korain Biotech Co., Ltd., Shanghai, China) according to the manufacturer’s instructions.

Serum syndecan-1 levels were analyzed using the SDC1 ELISA kit (catalog #E3344Hu). The intra-assay coefficient of variation (CV) of Syndecan-1 was <8% and the inter-assay CV was <10% for this parameter. The serum concentration of heparan sulfate was analyzed using the HS/HPS ELISA Kit (catalog #E9005Hu). The intra-assay CV of heparan sulfate was <10% and the inter-assay CV was <12% for this parameter. The results are expressed in ng mL^-1^.

### Randomization and Blinding

Before anaesthesia induction, the patients were randomly allocated to sevoflurane-based or desflurane-based anaesthesia using the closed envelope method. The anaesthesia and surgery team and the statistician were blinded to the study goals. A data collector who was not involved in the study coded the blood samples and collected patient data. The research team members were blinded to the allocation until the statistical analysis was completed.

### Statistical Analysis

Data were analyzed using the Statistical Package for the Social Sciences (SPSS) software (IBM SPSS Statistics for Windows, Version 25.0. Armonk, NY: IBM Corp., 2017). Percentage and frequency values were used for categorical variables, and the median, minimum, and maximum [med. (min.-max.)] were presented for quantitative data. The chi-square test was used to compare the two qualitative variables. The comparison of the two continuous variables was analyzed using the Mann-Whitney U test. Friedman variance analysis was performed for repeated measures of dependent variables. The Wilcoxon signed rank test was used for pairwise comparisons if statistical significance was found in the variance analysis. The type I error rate (α) was taken as 0.05 in the study.

The sample size was determined according to the change in mean heparan sulfate levels in the postoperative period based on the findings of a previously published study (allocation 1:1, two-sided).^1^ Accordingly, 22 patients per group were needed to obtain a 0.80 power with a large effect size and an alpha error of 0.05.

## Results

**Figure 1:**
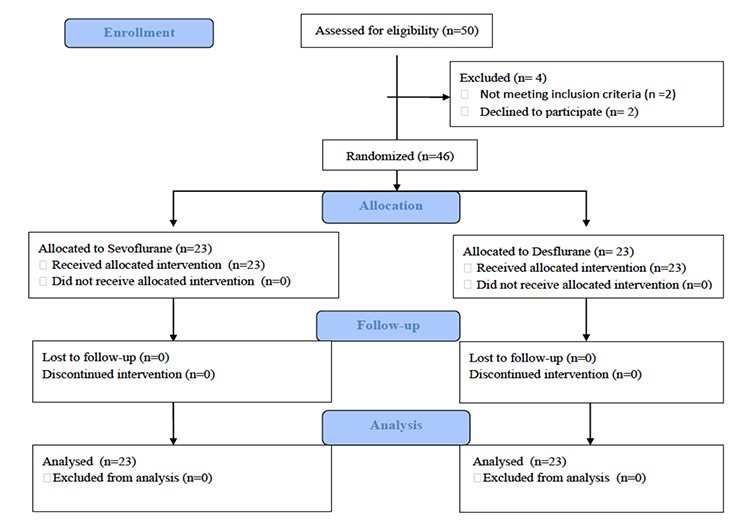
Flowchart of the study.

By considering possible dropouts, 50 patients were asked to participate in the study. Two patients declined to participate, and the other two were excluded from the study because of protocol violations (blood transfusion and conversion to laparotomy). Finally, data from 46 patients were analyzed (Figure 1).

The demographic and surgical characteristics of the participants in both groups were similar (Table 1). None of the patients received blood products. There was no intraoperative hypotensive period in any patient. The body temperature remained >36 ºC during the surgery.

**Figure 2:**
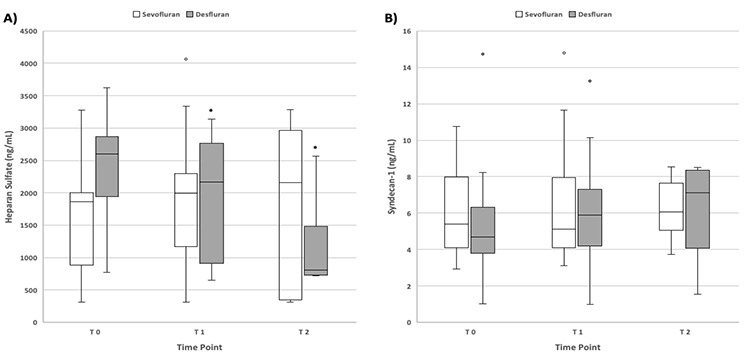
Alteration of heparan sulfate and syndecan-1 levels at the time points. Box and whisker plots of heparan sulfate (A) and syndecan-1 (B) according to sevoflurane and desflurane anaesthesia before induction (T_0_), 5 min after pneumoperitoneum (T_1_), and 5 min after extubation of the endotracheal tube (T_2_). The bottom and top of each box represent the 25^th^ and 75^th^ percentiles, respectively, the line inside the box is the median, the bottom and top of whiskers show the minimum and maximum range, respectively, and open circles indicate the outliers. *Significant difference (*P* < 0.05) in heparan sulfate levels in the desflurane group compared with the values at T_0_ and T_1_ time points.

There was no statistical difference between the sevoflurane and desflurane groups in terms of heparan sulfate and syndecan-1 levels at any time point (Figure 2). A statistically significant difference was found only in the desflurane group in the intragroup comparisons of the measurements of heparan sulfate levels (χ^2^=29.826, *P* < 0.001). Further analysis of the matched pairs of the time points showed that *P*=0.036 (Z=-2.099) for T_1_-T_0_, *P* < 0.001 (Z=-3.924) for T_2_-T_0_, and < 0.001 (Z=-4.197) for T_2_-T_1_ (Figure 2). There was no significant difference at any time point in syndecan-1 levels between the desflurane and sevoflurane groups.

**Figure 3:**
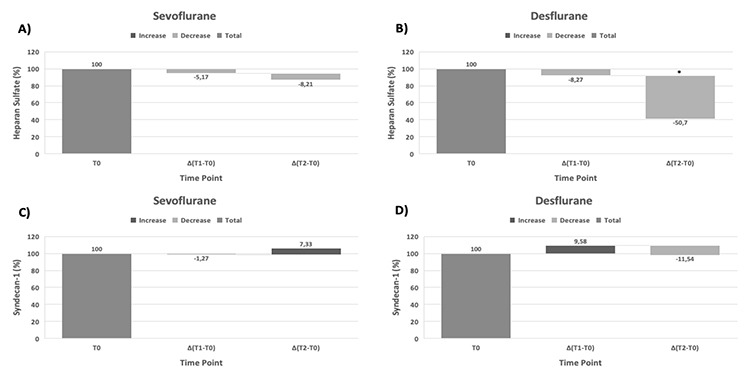
Comparison of the change percentage of heparan sulfate and syndecan-1. The waterfall chart shows the change in the percent of heparan sulfate (A), (B), and syndecan-1 (C), (D) from the baseline measurement at T_1_ and T_2_ time points according to sevoflurane and desflurane anaesthesia. *Significant difference (*P* < 0.05) in heparan sulfate change percentage in the desflurane group between T_2_ and T_1_.

A final analysis was performed to determine the difference in the change percentage of syndecan-1 and heparan sulfate at the time points. Accordingly, the change percent between the T_2_ and T_1_ of heparan sulfate in the desflurane group was found to be significantly higher than that in the sevoflurane group (*P*=0.034) (Figure 3).

## Discussion

The results of this study illustrated that heparan sulfate levels had decreased gradually in the desflurane group, indicating that desflurane had more protective effects on the endothelium than sevoflurane.

Many factors, such as major trauma and surgery, lead to endothelium damage and change endothelial functions.^6^ Surgical stress causes the release of cathepsin B, a lysosomal protease stored in endothelial cells, and subsequent degradation of the glycocalyx.^17^ Sevoflurane exerts a protective effect on the endothelial glycocalyx by stabilizing the lysosomal membrane.^18,19^ and suppressing the proinflammatory agents responsible for inducing lysosomal discharge.^20^

Annecke et al.^11^ in an animal study using electron microscopy, we demonstrated that heparan sulfate, syndecan, and cathepsin B release increased after ischemia, in addition to a massive increase in endothelial glycocalyx degradation. They also observed that these adverse effects due to ischemia were attenuated after sevoflurane administration. Similarly, another experimental study reported that endothelial glycocalyx components increased in serum after ischemia/reperfusion; this increase was more prominent with sevoflurane than with propofol-based anaesthesia.^20^

Although sevoflurane was emphasized to have protective effects on the endothelial glycocalyx in previous experimental studies on animals, the results in human studies were contradictory. Kim et al.^9^ found that syndecan levels increased after laparoscopic surgery, indicating endothelial glycocalyx impairment. In addition, the authors stated that the increase in syndecan levels was more pronounced with sevoflurane than with propofol. Similarly, another study comparing the effects of sevoflurane and propofol against ischemia/reperfusion damage in patients who underwent knee surgery concluded that sevoflurane did not have a protective effect on the endothelial glycocalyx.^14^ In contrast, Fang et al.’s^21^ study’s results denoted the endothelial protective effects of sevoflurane in cardiac surgery patients. Our study results can also be interpreted as suggesting that sevoflurane may have protective effects on the endothelium because the serum syndecan-1 and heparan sulfate levels remained unchanged.

Decomposition of syndecan from the endothelial glycocalyx structure requires protease activity, whereas lyase heparinase activity is required to degrade heparan sulfate in humans.^22^ Our findings showed no evident alteration in syndecan-1 levels with desflurane, but heparan sulfate levels significantly decreased compared with baseline values. These findings can be interpreted as desflurane preventing endothelial glycocalyx degradation by affecting lyase heparinase activity and incorporating heparan sulfate molecules into the glycocalyx structure. In the literature, very few studies have examined desflurane’s effects on endothelial glycocalyx. Contrary to our results, Oh et al.^15^ found no difference between desflurane and propofol in protecting the endothelial glycocalyx from ischemia/reperfusion injury knee arthroplasty patients. The main difference between Oh et al.^15^ and our study was that the mean age of the participants was higher in the trial conducted by Oh et al.^15^ than in our patients. The endothelial glycocalyx structure becomes more prone to deterioration with advanced age. Therefore, we can hypothesize that the protective effect of desflurane may be obscured by age.

Although syndecan-1 levels were similar between desflurane and sevoflurane, the results of our study suggested that desflurane was more effective than sevoflurane in protecting endothelial glycocalyx integrity, depending on the obvious decrease in heparan sulfate values. Although injury and protection of the endothelium are multifactorial, there is clear evidence that anaesthetics are somehow involved in this process. Despite these findings, there is a need for large-scale human studies to reveal more definitive results because there are conflicting studies on sevoflurane and very limited studies on desflurane in the literature.

### Study Limitations

The main limitation of this study is the lack of a control group comprising total intravenous anaesthesia. In addition, the study depicts endothelial glycocalyx degradation by measuring its components in plasma instead of directly visualizing the endothelial glycocalyx structure with electron microscopy. Furthermore, another limitation is that the enzyme levels that affect endothelial glycocalyx are not included. We believe that the analysis of these enzymes will further support our results. Finally, early postoperative pain levels were not evaluated in this study. Although inflammation can lead to pain, it does not always accompany overt inflammation.^23^

## Conclusion

The damage caused by surgical stress on the endothelial glycocalyx can be reduced by both desflurane and sevoflurane. However, the protective effect of desflurane is more prominent than that of sevoflurane.

## Figures and Tables

**Table 1:**
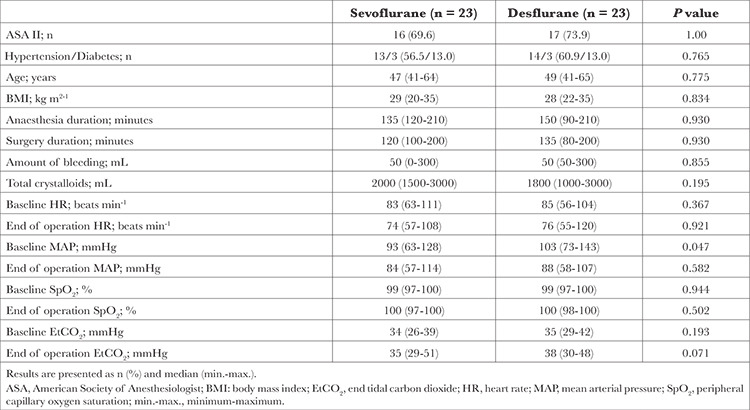
Demographic and Surgical Characteristics of Patients Randomized to Sevoflurane and Desflurane Groups
